# Characterization of Cutaneous Bacterial Microbiota from Superficial Pyoderma Forms in Atopic Dogs

**DOI:** 10.3390/pathogens9080638

**Published:** 2020-08-06

**Authors:** Caitlin E. Older, Aline Rodrigues Hoffmann, Kathleen Hoover, Frane Banovic

**Affiliations:** 1 Department of Veterinary Pathobiology, College of Veterinary Medicine & Biomedical Sciences, Texas A&M University, College Station, TX 77843, USA; c.older@tamu.edu; 2 Department of Small Animal Medicine and Surgery, College of Veterinary Medicine, University of Georgia, Athens, GA 30602, USA; khoover@uga.edu

**Keywords:** canine pyoderma, *Staphylococcus*, microbiota, bacterial folliculitis, epidermal collarette, skin, bacteria

## Abstract

Although *Staphylococcus pseudintermedius* is considered the major pathogen associated with superficial canine pyoderma, no study has investigated the entire bacterial community in these lesions with molecular techniques. The objectives of this study were to characterize the bacterial microbiota in two forms of superficial canine pyoderma lesions, superficial bacterial folliculitis (SBF) and epidermal collarette (EC), especially in terms of the staphylococcal community. Swabs from 12 SBF and 9 EC lesions were obtained from eight and six atopic dogs, respectively. Eight samples from the axilla and groin of four healthy dogs served as controls. DNA was extracted for 16S rRNA gene sequencing and quantitative polymerase chain reaction of *Staphylococcus* spp. and *S. pseudintermedius*. Healthy skin samples harbored significantly more diverse bacterial communities than pyoderma samples. Healthy samples had communities that were more similar to each other, and were distinct from pyoderma samples. *Staphylococcus* spp. abundance was increased in pyoderma samples, especially those from EC samples. Although determining species-level identities of staphylococcal sequences revealed many species, *S. pseudintermedius* was the primary staphylococcal species found in all sample types. As expected, there are many differences in the microbiota when comparing healthy and canine pyoderma lesions samples. These lesions do not seem to be associated with a change in the relative abundance of specific *Staphylococcus* species, but simply an overall increase in *Staphylococcus* spp. abundance. The results of this study provide a starting point for future studies investigating how antimicrobial treatments may further change the microbiota associated with these lesions.

## 1. Introduction

Canine superficial pyoderma, defined as a superficial bacterial infection of the epidermis and hair follicle [1], is a common diagnosis with a prevalence of up to 10–15% in private practices within the United States [2,3]. Primary superficial bacterial pyoderma in dogs is uncommon and most cases of recurrent superficial pyoderma in dogs are associated with an underlying allergic disease (e.g., atopic dermatitis, cutaneous adverse food reactions). Typical clinical manifestations of canine superficial pyoderma include three distinct forms: superficial bacterial folliculitis (SBF), bullous impetigo (BI), and epidermal collarettes (EC). Traditionally, epidermal collarettes have been thought to be a later stage of a superficial bacterial folliculitis (SBF) lesion, developing after pustules have ruptured and leaving the characteristic crater-like lesion [4,5]. However, recent research has revealed epidermal collarettes to have a unique histopathological phenotype, potentially attributed to a different pathology involving digestion of corneodesmosomes, resulting in epidermal splitting. Although previously, this epidermal splitting was thought to be due to exfoliative toxins produced by *Staphylococcus (S.) pseudintermedius* [6], the genes for these toxins were not found in a recent analysis of *S. pseudintermedius* isolates from epidermal collarettes, leaving the mechanism of this exfoliation still not well understood [7].

While *S. pseudintermedius* is the major pathogen associated with canine superficial pyoderma lesions, other bacteria can also be responsible. Furthermore, the presence of *S. pseudintermedius* does not signal an infection, since we know this is a commensal on canine skin [1,8,9]. Instead, an increase in abundance of *S. pseudintermedius* is characteristic of infection [8]. When infections are not properly treated, *S. pseudintermedius* can develop resistance, making clearing the infection even more difficult and costly [5].

All previous studies characterizing the microbial aspects of canine pyodermas have been culture-based [8,10,11,12,13]. Molecular techniques, such as next-generation sequencing (NGS), enable a more comprehensive characterization of the changing cutaneous microbiota with disease and its relationship to clinical features. Next-generation sequencing of the bacterial communities of healthy and atopic canine skin have been performed [14,15], however, to our knowledge, there are no studies on the microbial communities present in superficial canine pyoderma lesions using this technique.

The goals of this study were to utilize two molecular techniques, NGS and quantitative polymerase chain reaction (qPCR), to describe the microbiota in superficial canine pyoderma lesions of atopic dogs. In addition to evaluating the bacterial diversity and composition, we also sought to describe the staphylococcal communities in these lesions.

## 2. Results

**Figure 1:**
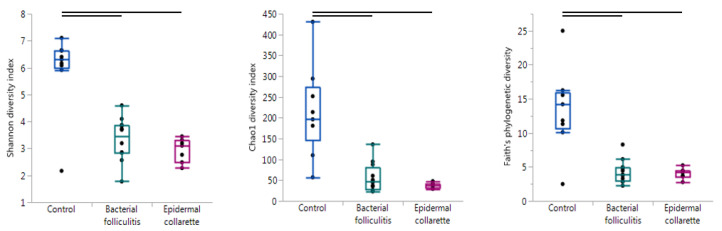
Evaluating alpha (within-sample) diversity. Using the Shannon diversity (Kruskal-Wallis test *p* = 0.0021), Chao1 diversity (*p* = 0.0002), and Faith’s Phylogenetic diversity indices (*p* = 0.0034), samples were found to be significantly different in diversity. In particular, control samples were more diverse than both the bacterial folliculitis and epidermal collarette samples (lines indicate pairwise tests resulting in *p* < 0.01).

In order to evaluate the overall diversity within each sample, alpha diversity was calculated using the Chao1 diversity index, Faith’s phylogenetic diversity, observed OTUs, Pielou’s evenness, and Shannon’s diversity index. These metrics evaluate diversity in slightly different ways, giving values that can be compared between different sample types to analyze diversity. A sample with a higher value for any of these metrics has higher diversity in terms of the number of different taxa (richness) and/or how evenly they are in abundance (evenness). The control samples were significantly more diverse than the SBF and EC samples with all metrics (Figure 1, Table 1). None of the alpha diversity metrics revealed significant differences between the SBF and EC samples.

**Figure 2:**
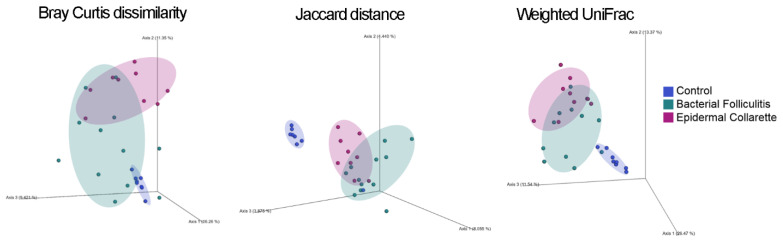
Evaluating beta (between-sample) diversity. In these plots, each dot represents a single sample, where dots that are closer together are more similar to each other in terms of the taxa that were found. Visually, the control samples cluster tightly and distinctly from the pyoderma samples, which also cluster somewhat uniquely within their lesion types. This unique clustering between the three groups was further supported by ANOSIM, which found significant dissimilarity with the Bray-Curtis dissimilarity (R = 0.6802), Jaccard distance (R = 0.7101), weighted (R = 0.6539), and unweighted UniFrac (R = 0.6302) metrics (all *p* = 0.001).

Beta diversity was also analyzed, which evaluates diversity by taking into account the different taxa that are present or absent across samples types and/or sometimes their relative abundance and phylogenetic relationships. The Bray-Curtis dissimilarity, Jaccard distance, and the weighted and unweighted UniFrac [16] matrices were used to evaluate clustering, followed by ANOSIM testing for significance. Again, significant differences were found between the control, the bacterial folliculitis and epidermal collarette samples (Bray-Curtis dissimilarity: R = 0.6802, Jaccard distance: R = 0.7101, weighted UniFrac: R = 0.6539, and unweighted UniFrac: R = 0.6302; all *p* = 0.001). This can be observed in Figure 2, where healthy samples cluster closely together and distinctly from both lesion sample types. The two different lesions also form somewhat unique clusters, however, these were not significantly different from each other (Table 1).

**Figure 3:**
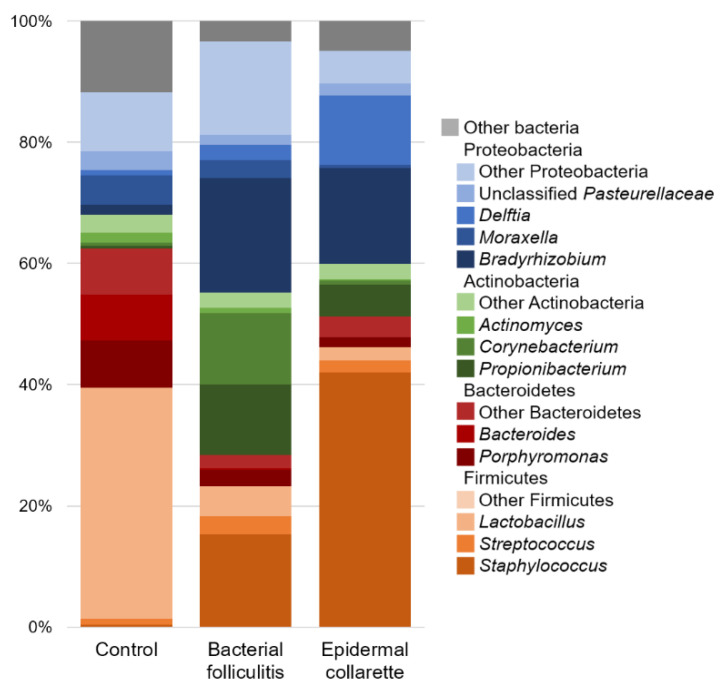
Average relative abundance of bacterial taxa. *Staphylococcus* spp. were found to be significantly different between the different sample types (*p* < 0.01), with canine pyoderma samples having much more of these bacteria. Although an increase in *Staphylococcus* spp. is found, the normal microbiota is still present in affected samples, but in lesser abundance.

**Figure 4:**
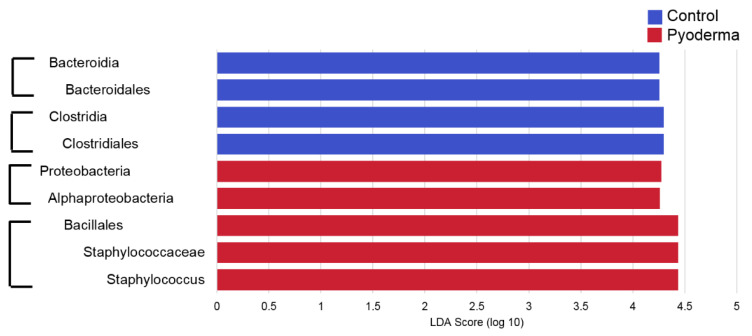
Linear discriminant analysis (LDA) effect size (LEfSe) results comparing control and pyoderma samples, with an LDA score (log 10) cut-off of 4. Brackets show taxa that are in the same taxonomic line. Importantly, *Staphylococcus* spp. were found to be increased in pyoderma samples, as was also shown in other analyses presented. More differentially abundant taxa were identified with a lower LDA score (log 10) and can be found in Supplementary Table S3.

**Figure 5:**
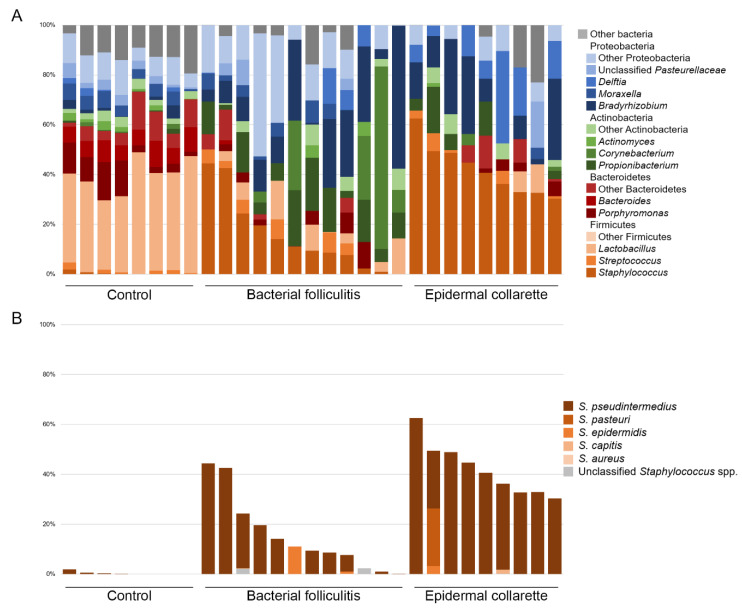
Relative abundance of bacterial taxa and *Staphylococcus* species by individual sample. (**A**) Samples taken from control dogs seem to be much more consistent in the relative abundance of taxa found relative to the two pyoderma sample types, however, a consistent increase in *Staphylococcus* spp. is seen across the pyoderma samples, especially those taken from epidermal collarettes. (**B**) When looking at the relative abundance of *Staphylococcus* species across samples, *S. pseudintermedius* is the primary species found. Some samples had very little or no *Staphylococcus* sequences.

There was a noticeable difference in the relative abundance of *Staphylococcus* spp. (Figure 3), with significantly higher relative abundance in samples from both canine pyoderma lesion types (*p* < 0.01, Figure 4), but especially the epidermal collarette samples (Linear discriminant analysis (LDA) score (log 10) = 4.625). Importantly, this significant finding of higher *Staphylococcus* spp. relative abundance in the EC samples was consistent across the individual samples (Figure 5A). Although we observed higher *Staphylococcus* spp. relative abundance in pyoderma samples, the microbiota on healthy canine skin, consisting of a variety of bacterial species, was still present but in different proportions. In addition to *Staphylococcus* spp., other taxa including *Propionibacterium* spp. (increased in both pyoderma lesion types), *Fusobacterium* spp. (increased in control), and *Bacteroides* spp. (increased in control) were also found to be differentially abundant (Supplementary Table S2).

**Figure 6:**
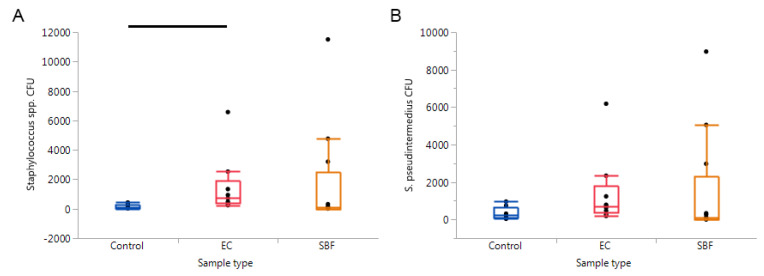
Results from quantitative PCRs targeting (**A**) *Staphylococcus* spp. and (**B**) *S. pseudintermedius*. Significant findings were only revealed when comparing the *Staphylococcus* spp. CFU between the control, epidermal collarette, and superficial bacterial folliculitis samples (*p* = 0.0140), with this significance being due to the pairwise comparison of control and epidermal collarette samples (*p* = 0.0053).

Further analysis of the *Staphylococcus* spp. sequences revealed *S. pseudintermedius* as the most abundant staphylococcal species across all samples types (Figure 5B). Relative abundances of all identified staphylococcal species can be found in Supplementary Table S4. Quantitative PCR for *Staphylococcus* spp. and *S. pseudintermedius* abundance revealed significant differences in the abundance of *Staphylococcus* spp. (*p* = 0.0140), but not *S. pseudintermedius* (*p* = 0.0819) between the three sample types (Figure 6). These differences in *Staphylococcus* spp. abundance could be attributed to the higher abundances in EC relative to control samples (*p* = 0.0053).

## 3. Discussion

In this study, a clear bacterial dysbiosis was demonstrated in canine pyoderma samples, with decreased diversity and decreased relative abundance of “normal” microbial inhabitants relative to samples from healthy canine skin. In terms of alpha diversity, samples taken from healthy dogs were significantly more diverse than samples from either pyoderma lesion type. Additionally, these control samples clustered separately from lesional samples with respect to beta diversity, indicating these different samples types are different in the taxa that are present and in their relative proportions. Although *Staphylococcus* spp. are often the only microorganism isolated from canine pyoderma lesions using aerobic bacterial cultures, the “normal” microbiota is still present, but in lesser abundance.

**Figure 7:**
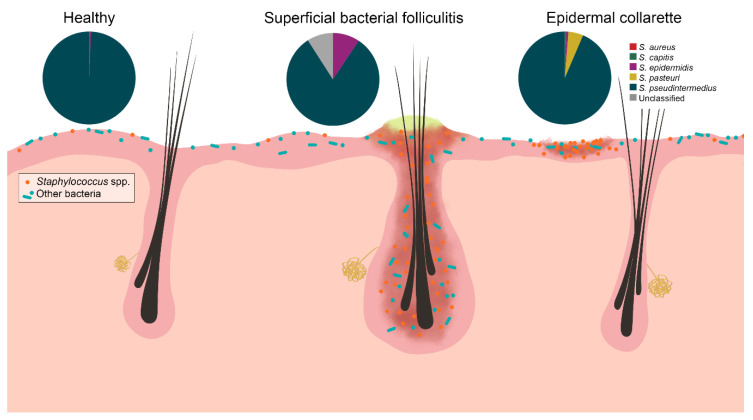
*Staphylococcus* spp. were increased in relative abundance in both pyoderma samples compared to controls, especially in the epidermal collarette samples. This finding may be attributed in part due to the more superficial phenotype of epidermal collarettes, which could allow for easier access to the increased numbers of *Staphylococcus* spp. Characterization of staphylococcal communities revealed *S. pseudintermedius* to be the most abundant *Staphylococcus* species in all sample types. There was no a significant difference in abundance of this or any other staphylococcal species.

Corroborating with findings of previous culture-based studies [8,10,11,12,13], significant differences in the abundance of *Staphylococcus* spp. were found between control and lesional samples. Additionally, significant differences were found between the lesions, with epidermal collarettes having increased proportions of *Staphylococcus* spp. Perhaps part of this observation can be attributed to sampling, since bacterial folliculitis pustules are smaller in terms of surface size relative to epidermal collarettes and the sampling methodology may not have collected the microbiota in the deeper hair follicle infundibulum. The main species identified in all sample types was *S. pseudintermedius*, with other species identified in lower relative abundances (Figure 7). These findings are consistent with a previous study, where similar *Staphylococcus* species were found between healthy dogs and those with atopic dermatitis (AD) flares, with significant differences in total *Staphylococcus* spp. abundance [14].

The changes in relative abundance of other taxa are also of interest, especially when they may potentially interact directly with *Staphylococcus* spp. to either inhibit or enhance its pathogenicity [17]. One taxon of interest is *Corynebacterium* spp., a genus that was previously reported to be increased in a murine model of human atopic dermatitis [18] and in the groin of dogs with naturally occurring canine atopic dermatitis (cAD) [14]. However, these bacteria’s role in dermatologic diseases is unclear [19]; its abundance increased after treatment in pediatric atopic dermatitis [20] and one species has been shown to actually prevent overgrowth of *Streptococcus pneumoniae* [19]. In the present study, increased abundance of *Corynebacterium* spp. was observed in four SBF samples (three samples from the same dog), but did not reach statistical significance and did not seem to be consistently increased with *Staphylococcus* spp. Perhaps species-level analysis would reveal changes in *Corynebacterium* species that may be more informative. Another taxon of interest in the cutaneous microbiota is *Propionibacterium* spp. (now *Cutibacterium* spp.; included under the genus *Propionibacterium* in this analysis). In this study, *Propionibacterium* spp. were increased in the SBF samples compared to the control samples and, when the two lesional types were combined, in the pyoderma samples compared to the control samples. However, the role of *Propionibacterium* spp. in healthy canine skin is still unknown, considering the varying results from previous studies regarding their abundance [14,21,22,23] and a potentially different sebum composition on canine skin, which could affect the growth of the bacteria [17,24].

Studies describing the synergistic or competitive relationships between *S. pseudintermedius* and other bacteria would aid in better understanding concurrent changes in abundance that are observed. In humans, *Staphylococcus* spp. are part of the normal microbiota, however, in healthy individuals, this is typically *S. epidermidis* that is replaced by *S. aureus* in atopic dermatitis [20,25]. A recent study by Nakatsuji et al. described the production of antimicrobial peptides by coagulase negative staphylococcal species *S. epidermidis* and *S. hominis* isolated from non-AD skin. These isolates demonstrated anti-*S. aureus* activity not only in culture, but also when applied to the skin of humans with AD [26]. Other studies have also identified anti-*S. aureus* actions by non-*S. aureus Staphylococcus* species [27,28,29]. In our study, *S. pseudintermedius* was the primary species found in both control and affected samples, with only low relative abundance of other species. These results suggest there is likely not another staphylococcal species that could play a protective role on canine skin, but perhaps another bacterial or fungal species is currently unknown to be important for maintaining a healthy community [30,31]. Whole genome sequencing for identification of strain-level resolution may better decipher the protective and harmful dynamics of staphylococcal communities.

Within this study, sequencing of the V1-3 region of the 16S rRNA gene was performed. This region has been described to more accurately represent the skin microbiota, while also allowing for differentiation of *Staphylococcus* species [32]. However, it is important to note that this region is not able to differentiate between all staphylococcal species. For example, comparing the NCBI Reference Sequence Database entries for this region of the sequence for *S. pseudintermedius*, the predominant species found on canine skin, to its close relatives *S. delphini* and *S. intermedius* indicates that they are identical. Therefore, in the future, studies should utilize culture-based methods or other sequencing regions for *Staphylococcus* spp. Within our sample cohort, phenotypic, biochemical, and genotypic analysis identified all bacterial isolates from the nine epidermal collarette lesions and six superficial bacterial folliculitis samples as *S. pseudintermedius*.

There are certainly other aspects of the canine pyoderma microbiota that should be further studied. These include deep pyodermas, which would likely have a more altered bacterial community, since these are less affected by the dog’s environment. Additionally, looking at recurrent/resistant infections, effect of therapy on the overall microbiota, and comparing topical and systemic therapies would allow better understanding of current therapeutics in terms of the whole microbial population and potentially allow for more specific treatment. Longitudinal studies involving treatment would reveal how long the microbiota takes to return to baseline and what kind of changes occur to resolve lesions and remain after remission. Considering this was a pilot study, there is a clear need for studies involving larger sample sizes. Furthermore, future studies including other lesions types, such as papules or crusts, may better describe the vast range of lesions that are seen with superficial canine pyoderma.

## 4. Materials and Methods

Dogs of any breed, body weight, and sex with active clinical lesions of SBF and/or EC were selected for this study; active lesions were defined by the presence of surrounding lesional erythema [7]. To be included in the study, dogs were not treated with antibacterial shampoos, systemic and/or topical antibiotics for at least 2 weeks prior to sampling. The study was approved by University of Georgia institutional animal care and use committee (study CR-459) and all the owners gave written informed consent before swab collection. Control samples were collected from four unrelated healthy client-owned Bluetick Coonhound dogs (two neutered males and two spayed females) with no history of skin disease. Samples from 12 SBF and nine EC lesions were taken from eight and six atopic dogs, respectively. The signalment of these dogs can be found in Supplementary Table S1.

Superficial pyoderma samples were collected from the axilla and groin skin lesions. For bacterial folliculitis samples, pustules were ruptured with a sterile needle after which Isohelix buccal swabs (Cell Projects Ltd., Kent, UK) and sterile bacterial culture swabs (BD BBL CultureSwab, Sparks, MD, USA) were applied. Marginal crusts of epidermal collarettes were gently lifted using sterile gloves, and the Isohelix buccal and sterile bacterial culture swabs were applied underneath the crusts. In addition to the pyoderma samples, two Isohelix buccal swabs samples (axilla and groin) were taken from four healthy dogs [33].

The bacterial culture swabs were inoculated onto Columbia agar with 5% sheep blood (Fisher Scientific, Waltham, MA, USA) using the four quadrants technique and incubated at 35 °C for 18–24 h. All isolates were identified phenotypically, biochemically, and through PCR amplification of *hsp60* and *nuc* gene, as described previously [34,35].

For Isohelix swabs samples, the swabs were put into an MO BIO PowerBead tube (MOBIO Laboratories, Carlsbad, CA, USA) and stored at 4 °C until DNA extraction using the MO BIO PowerSoil DNA Isolation Kit with a modified protocol. Extracted DNA was then stored at −80 °C until further processing.

DNA extracted from the skin swabs was sequenced on an Illumina MiSeq (Illumina Inc., San Diego, CA, USA) utilizing primers targeting the V1-3 region of the 16S rRNA gene (V1_27F: AGAGTTTGATCMTGGCTCAG, V3_534R: ATTACCGCGGCTGCTGG) at the University of Minnesota Genomics Center (Minneapolis, MN, USA). In addition to the DNA extracted from the samples, controls were also sequenced. Operational taxonomic units (OTUs) identified as contaminants, as previously described [36], were removed, in addition to *Ralstonia* spp., which have been shown to be a common reagent contaminant [37]. Raw sequences are accessible under BioProject ID PRJNA478200 in the NCBI Sequence Read Archive.

Resulting sequences first had primers removed with cutadapt, were demultiplexed in QIIME 1.9.1, and then, processed in QIIME2 (February 2017 release) [38]. Sequence dereplication and chimera removal were performed with vsearch [39] and uchime [40], and taxonomic assignments were determined using the Greengenes database (13_8 release) [41] with a scikit-learn classifier [42]. Prior to diversity analyses, data were rarefied. In addition to the standard microbiota analysis (alpha diversity, beta diversity, taxonomic summary, differential taxa abundance testing), *Staphylococcus* spp. sequences were furthered analyzed through extraction and alignment to a *Staphylococcus* spp. reference sequence alignment [32], which was modified to include sequences from staphylococcal species identified since 2012: *S. argensis, S. argentus, S. cornubiensis, S. edaphicus, S. debuckii, S. jettensis, S. petrasii, S. pseudoxylosus, S. schweitzeri* and *S. stepanovicii.* These sequences were obtained from the NCBI Reference Sequence Database and the Ribosomal Database Project. The reference alignment was trimmed to the sequencing region and utilized for classification in QIIME2 via BLAST+ [43], where those with <99% identity to a species were considered unclassified.

Extracted DNA was also used for quantitative PCR (qPCR) targeting *Staphylococcus* spp. [44] and *S. pseudintermedius* [15]. A culture-derived standard curve of *S. pseudintermedius* was used for both PCRs, allowing for absolute quantification. Reactions were run on a Bio-Rad CFX Connect (Bio-Rad Laboratories, Hercules, CA, USA) as previously described [33]. Resulting data were analyzed in CFX manager™ (Bio-Rad Laboratories) and normalized to DNA concentration based on the Qubit High Sensitivity dsDNA assay (Invitrogen, Carlsbad, CA, USA).

Next-generation sequencing and qPCR results were analyzed using JMP Pro 13 (SAS Institute, Cary, NC, USA), where normality of data was tested using the Shapiro–Wilk test, followed by hypothesis testing with Kruskal–Wallis tests, since data were not normally distributed. Furthermore, NGS data were analyzed using the ANOSIM test in R with the vegan package and the Linear Discriminant Analysis (LDA) Effect Size (LEfSe) algorithm [45]. For Wilcoxon and Kruskal–Wallis tests for differential abundance, taxonomy tables were filtered to include only genera present at greater than 1% in at least three samples, allowing the evaluation of 42 taxa.

## 5. Conclusions

In this study, next-generation sequencing revealed superficial canine pyodermas to have a unique bacterial microbiota with marked dysbiosis relative to healthy skin. These communities have significantly higher abundances of *Staphylococcus* spp., with no apparent changes in the proportions of this or any other *Staphylococcus* species. Importantly, the “normal” microbiota is still present in pyoderma samples, but in much lesser abundance. Additionally, differences between superficial bacterial folliculitis and epidermal collarette lesions were identified, although perhaps are not contributing to the different phenotypes, but instead a result of the differences in innate features of the lesions. Future studies on the effect of therapy and characterizing lesions at the strain level may allow for the development of more specific treatment regimens.

## Figures and Tables

**Table 1:** Results from Wilcoxon and Kruskal-Wallis tests on alpha diversity data and analysis of similarities (ANOSIM) tests on beta diversity data. EC—epidermal collarette; SBF—superficial bacterial folliculitis.

	Control vs. Pyoderma	Control vs. EC	Control vs. SBF	EC vs. SBF
**Alpha diversity**				
Chao1 diversity index	<0.001	0.001	<0.001	0.337
Faith’s phylogenetic diversity	<0.001	0.009	0.008	0.915
Observed OTUs	<0.001	0.007	0.003	1.000
Pielou’s evenness	<0.001	0.007	0.012	0.145
Shannon diversity index	<0.001	0.001	<0.001	0.337
**Beta diversity**				
Bray-Curtis	R = 0.908, *p* = 0.001	R = 1.000, *p* = 0.002	R = 0.923, *p* = 0.001	R = 0.168, *p* = 0.030
Jaccard	R = 0.923, *p* = 0.001	R = 0.959, *p* = 0.001	R = 0.983, *p* = 0.001	R = 0.154, *p* = 0.023
Unweighted UniFrac	R = 0.956, *p* = 0.001	R = 0.974, *p* = 0.001	R = 0.966, *p* = 0.001	R = 0.023, *p* = 0.321
Weighted UniFrac	R = 0.866, *p* = 0.001	R = 1.000, *p* = 0.001	R = 0.854, *p* = 0.001	R = 0.135, *p* = 0.055

## Supplementary Materials

The following are available online at https://www.mdpi.com/2076-0817/9/8/638/s1, Table S1: Signalment of sample cohort, Table S2: Average relative abundance of bacterial taxa and p values from Wilcoxon and Kruskal-Wallis test comparing average abundance, Table S3: LEfSe results comparing control and canine pyoderma samples, Table S4: Relative abundance (relative to staphylococcal sequences) of staphylococcal species.
