# Supplementary material for: Characterization of Cutaneous Bacterial Microbiota from Superficial Pyoderma Forms in Atopic Dogs
Source: Pathogens. 2020 Aug 6;9(8):638. doi: 10.3390/pathogens9080638 (PMC7459807; doi:10.3390/pathogens9080638)
Supplement: Supplementary file 1 [file pathogens-09-00638-s001.pdf]

pathogens

MDPI

Table S1: Signalment of sample cohort. SBF = superficial bacterial folliculitis, EC = epidermal collarette.

Sample type	Sample label	Breed	Age	Sex	Disease	Antibiotic Usage (within Last 6 Months)	Other Medications
SBF	D1Fol1	Staffordshire Bull Terrier	3 years	SF	Atopic dermatitis	None	None
SBF	D1Fol2						
SBF	D1Fol3						
SBF	D2Fol	Great Pyrenees	2 years	SF	Atopic dermatitis	None	None
SBF	D3Fol	Vizsla	3 years	CM	Atopic dermatitis	Chloramfenicol	Oclactinib
EC	D4Col	American Pit Bull Terrier	10 years	SF	Atopic dermatitis	None	None
SBF	D4Fol1						
SBF	D4Fol2						
EC	D5Col1	Boston Terrier	10 years	SF	Atopic dermatitis	Cefpodoxime	None
EC	D5Col2						
SBF	D5Fol						
EC	D6Col	Labrador retriever	6 years	SF	Atopic dermatitis	None	None
SBF	D6Fol						
SBF	D7Pus1	Mixed breed	1 year	CM	Atopic dermatitis	None	None
SBF	D7Pus2						
SBF	D8Pus	Cocker Spaniel	8 years		Atopic dermatitis	None	None
EC	D9Col1	Mastiff	1 year	M	Atopic dermatitis	None	None
EC	D9Col2						
EC	D10Col1	Yorkshire terrier	9 years	M	Atopic dermatitis	Clindamycin	None
EC	D10Col2						
EC	D11Col	Mixed breed	5 years	SF	Atopic dermatitis	Marbofloxacin	Oclactinib
Control	Baxilla	Bluetick coonhound	2 years	CM	None	None	None
	Bgroin						
Control	Haxilla	Bluetick coonhound	2 years	CM	None	None	None
	Hgroin						
Control	Jaxilla	Bluetick coonhound	2 years	SF	None	None	None
	Jgroin						
Control	Raxilla	Bluetick coonhound	2 years	SF	None	None	None
	Rgroin						

Table S2. Average relative abundance of bacterial taxa and p-values from Wilcoxon and Kruskal-Wallis test comparing average abundance. Average, median (min-max). EC = epidermal collarette, SBF = superficial bacterial folliculitis. Pyoderma column includes data from both EC and SBF samples.

Phylum	Class	Order	Family	Genus	Control vs Pyoderma	Control vs EC vs SBF	Control	Epidermal Collarette	Superficial Bacterial Folliculitis	Pyoderma
Actinobacteria	Actinobacteria	Actinomycetales	Actinomycetaceae	Actinomyces	0.005	0.012	0.02, 0.02 (0–0.03)	0, 0 (0–0.01)	0.01, 0 (0–0.06)	0.01, 0 (0–0.06)
			Corynebacteriaceae	Corynebacterium	0.673	0.376	0.01, 0 (0-0.02)	0.01, 0 (0–0.05)	0.12, 0 (0–0.73)	0.07, 0 (0–0.73)
			Micrococcaceae	Rothia	0.941	0.816	0, 0 (0–0.01)	0.01, 0 (0–0.06)	0.01, 0 (0–0.04)	0.01, 0 (0–0.06)
			Propionibacteriaceae	Propionibacterium	0.009	0.004	0, 0 (0–0.02)	0.05, 0.03 (0–0.19)	0.12, 0.12 (0.02–0.23)	0.09, 0.06 (0–0.23)
	Coriobacteriia	Coriobacteriales	Coriobacteriaceae	Collinsella	0	0.001	0.02, 0.02 (0–0.03)	0, 0 (0–0)	0.01, 0 (0–0.08)	0, 0 (0–0.08)
Bacteroidetes	Bacteroidia	Bacteroidales	Unclassified	Unclassified	0.001	0.004	0.02, 0.01 (0–0.05)	0, 0 (0–0)	0, 0 (0–0)	0, 0 (0–0)
			Paraprevotellaceae	Prevotella	0	0.001	0.01, 0.01 (0–0.02)	0, 0 (0–0.01)	0, 0 (0–0.06)	0, 0 (0–0.06)
			Bacteroidaceae	Bacteroides	0	0	0.08, 0.06 (0.06–0.11)	0, 0 (0–0)	0, 0 (0–0.02)	0, 0 (0–0.02)
			Porphyromonadaceae	Porphyromonas	0.015	0.049	0.08, 0.07 (0.02–0.15)	0.02, 0 (0-0.06)	0.03, 0.01 (0-0.11)	0.02, 0 (0-0.11)
			Prevotellaceae	Prevotella	0.003	0.011	0.03, 0.02 (0–0.06)	0, 0 (0-0)	0, 0 (0-0)	0, 0 (0-0)
	Flavobacteriia	Flavobacteriales	Flavobacteriaceae	Capnocytophaga	0.003	0.012	0.01, 0.01 (0–0.02)	0.01, 0 (0-0.13)	0, 0 (0-0.02)	0.01, 0 (0-0.13)
Firmicutes	Bacilli	Bacillales	Staphylococcaceae	Staphylococcus	0	0	0, 0 (0–0.02)	0.42, 0.41 (0.3-0.63)	0.15, 0.1 (0-0.44)	0.27, 0.3 (0-0.63)
		Gemellales	Gemellaceae		0.695	0.833	0, 0 (0–0)	0.01, 0 (0-0.06)	0.01, 0 (0-0.05)	0.01, 0 (0-0.06)
		Lactobacillales	Lactobacillaceae	Lactobacillus	0	0	0.03, 0.03 (0.02–0.06)	0, 0 (0-0)	0, 0 (0-0.02)	0, 0 (0-0.02)
			Streptococcaceae	Streptococcus	0.944	0.882	0.01, 0.01 (0–0.03)	0.02, 0.01 (0-0.07)	0.03, 0.01 (0-0.08)	0.02, 0.01 (0-0.08)
		Turicibacterales	Turicibacteraceae	Turicibacter	0	0	0.07, 0.05 (0.03–0.11)	0, 0 (0-0)	0, 0 (0-0)	0, 0 (0-0)
	Clostridia	Clostridiales	Unclassified	Unclassified	0	0	0.05, 0.05 (0.01–0.08)	0, 0 (0-0)	0, 0 (0-0)	0, 0 (0-0)
			Clostridiaceae	Clostridium	0	0	0.06, 0.06 (0.04–0.1)	0, 0 (0-0)	0, 0 (0-0)	0, 0 (0-0)
			Clostridiaceae	SMB53	0	0.001	0.01, 0.01 (0–0.02)	0, 0 (0-0)	0, 0 (0-0)	0, 0 (0-0)
			Lachnospiraceae	Blautia	0	0	0.02, 0.02 (0–0.04)	0, 0 (0-0)	0, 0 (0-0)	0, 0 (0-0)
			Peptostreptococcaceae		0.005	0.016	0.01, 0.01 (0–0.01)	0, 0 (0-0.04)	0, 0 (0-0.01)	0, 0 (0-0.04)
		Clostridiales	Veillonellaceae	Phascolarctobacterium	0	0	0.01, 0.01 (0–0.02)	0, 0 (0-0)	0, 0 (0-0)	0, 0 (0-0)
	Erysipelotrichi	Erysipelotrichales	Erysipelotrichaceae		0	0	0.01, 0.01 (0–0.02)	0, 0 (0-0)	0, 0 (0-0)	0, 0 (0-0)
			Erysipelotrichaceae	Eubacterium	0	0	0.03, 0.03 (0–0.06)	0, 0 (0-0)	0, 0 (0-0)	0, 0 (0-0)
			Erysipelotrichaceae	Catenibacterium	0.001	0.004	0.01, 0.01 (0–0.05)	0, 0 (0-0)	0, 0 (0-0)	0, 0 (0-0)
Fusobacteria	Fusobacteriia	Fusobacteriales	Fusobacteriaceae	Unclassified	0	0	0.02, 0.02 (0–0.06)	0, 0 (0-0)	0, 0 (0-0)	0, 0 (0-0)
			Fusobacteriaceae	Cetobacterium	0	0	0.01, 0.01 (0–0.03)	0, 0 (0-0)	0, 0 (0-0)	0, 0 (0-0)
			Fusobacteriaceae	Fusobacterium	0	0	0.05, 0.05 (0.01–0.1)	0, 0 (0-0.04)	0, 0 (0-0.03)	0, 0 (0-0.04)
Proteobacteria	Alphaproteobacteria	Rhizobiales	Bradyrhizobiaceae	Bradyrhizobium	0.002	0.006	0.02, 0.01 (0–0.05)	0.16, 0.13 (0-0.33)	0.19, 0.12 (0.01-0.58)	0.18, 0.13 (0-0.58)
		Sphingomonadales	Sphingomonadaceae	Sphingomonas	0.088	0.098	0, 0 (0–0.01)	0, 0 (0-0)	0.01, 0 (0-0.06)	0, 0 (0-0.06)
	Betaproteobacteria	Burkholderiales	Comamonadaceae	Delftia	0.627	0.012	0.01, 0 (0–0.03)	0.11, 0.07 (0-0.37)	0.03, 0 (0-0.14)	0.06, 0 (0-0.37)
		Neisseriales	Neisseriaceae		0.03	0.059	0, 0 (0–0.01)	0.02, 0 (0-0.1)	0, 0 (0-0)	0.01, 0 (0-0.1)
			Neisseriaceae	Conchiformibius	0.297	0.144	0.01, 0.01 (0–0.01)	0, 0 (0-0.01)	0.03, 0 (0-0.18)	0.02, 0 (0-0.18)
	Gammaproteobacteria	Pasteurellales	Pasteurellaceae	Unclassified	0.266	0.237	0, 0 (0–0)	0, 0 (0-0)	0.01, 0 (0-0.06)	0.01, 0 (0-0.06)
			Pasteurellaceae		0.009	0.024	0.03, 0.03 (0.01–0.06)	0.02, 0 (0-0.18)	0.02, 0 (0-0.1)	0.02, 0 (0-0.18)
		Pseudomonadales	Moraxellaceae	Enhydrobacter	0.002	0.005	0.02, 0.01 (0–0.05)	0, 0 (0-0)	0.01, 0 (0-0.04)	0, 0 (0-0.04)
			Moraxellaceae	Moraxella	0.019	0.012	0.05, 0.05 (0.01–0.07)	0.01, 0 (0-0.05)	0.03, 0.02 (0-0.09)	0.02, 0 (0-0.09)

			Moraxellaceae	Psychrobacter	0	0	0.01, 0.01 (0–0.02)	0, 0 (0–0)	0, 0 (0-0)	0, 0 (0-0)
			Pseudomonadaceae	Pseudomonas	0.622	0.861	0, 0 (0–0.01)	0.01, 0 (0–0.06)	0.01, 0 (0-0.04)	0.01, 0 (0-0.06)
Spirochaetes	Spirochaetes	Spirochaetales	Spirochaetaceae	Treponema	0.036	0.109	0, 0 (0–0.02)	0, 0 (0–0)	0, 0 (0-0.04)	0, 0 (0-0.04)
SR1					0	0.001	0.01, 0.01 (0–0.03)	0, 0 (0–0)	0, 0 (0-0)	0, 0 (0-0)
Tenericutes	Mollicutes	Mycoplasmatales	Mycoplasmataceae	Mycoplasma	1	0.843	0, 0 (0–0.02)	0.03, 0 (0–0.13)	0.03, 0 (0-0.16)	0.03, 0 (0-0.16)

Table S3. Linear discriminant analysis (LDA) effect size (LEfSe) results comparing control and canine pyoderma samples. $p < 0.01$ and LDA score (log 10) cut-off of 3.0.

Taxon.	Group with Higher Abundance	LDA Score (log 10)
Firmicutes. Bacilli. Bacillales. Staphylococcaceae. Staphylococcus	Pyoderma	4.435897
Firmicutes. Bacilli. Bacillales	Pyoderma	4.435882
Firmicutes. Bacilli. Bacillales. Staphylococcaceae	Pyoderma	4.435847
Firmicutes. Clostridia. Clostridiales	Control	4.296997
Firmicutes. Clostridia	Control	4.296997
Proteobacteria	Pyoderma	4.27615
Proteobacteria. Alphaproteobacteria	Pyoderma	4.259567
Bacteroidetes. Bacteroidia. Bacteroidales	Control	4.256126
Bacteroidetes. Bacteroidia	Control	4.256126
Bacteroidetes	Control	4.248844
Proteobacteria. Alphaproteobacteria.Rhizobiales	Pyoderma	4.247719
Proteobacteria. Alphaproteobacteria. Rhizobiales. Bradyrhizobiaceae	Pyoderma	4.230589
Proteobacteria. Alphaproteobacteria. Rhizobiales. Bradyrhizobiaceae. Bradyrhizobium	Pyoderma	4.230579
Actinobacteria. Actinobacteria. Actinomycetales	Pyoderma	4.186135
Actinobacteria. Actinobacteria	Pyoderma	4.186135
Fusobacteria	Control	3.94413
Fusobacteria. Fusobacteriia	Control	3.943757
Fusobacteria. Fusobacteriia. Fusobacteriales	Control	3.943237
Fusobacteria. Fusobacteriia. Fusobacteriales. Fusobacteriaceae	Control	3.937283
Actinobacteria. Actinobacteria. Actinomycetales. Propionibacteriaceae. Propionibacterium	Pyoderma	3.922449
Actinobacteria. Actinobacteria. Actinomycetales. Propionibacteriaceae	Pyoderma	3.922426
Bacteroidetes. Bacteroidia. Bacteroidales. Bacteroidaceae. Bacteroides	Control	3.889137
Bacteroidetes. Bacteroidia. Bacteroidales. Bacteroidaceae	Control	3.889045
Firmicutes. Clostridia. Clostridiales. Clostridiaceae	Control	3.880081
Firmicutes. Bacilli. Turicibacterales	Control	3.792753
Firmicutes. Bacilli. Turicibacterales. Turicibacteraceae. Turicibacter	Control	3.792752
Firmicutes. Bacilli. Turicibacterales. Turicibacteraceae	Control	3.792751
Firmicutes. Clostridia. Clostridiales. Clostridiaceae. Clostridium	Control	3.792542
Proteobacteria. Gammaproteobacteria. Pseudomonadales. Moraxellaceae	Control	3.786494
Firmicutes. Erysipelotrichi. Erysipelotrichales. Erysipelotrichaceae	Control	3.766502
Firmicutes. Erysipelotrichi	Control	3.766493
Firmicutes. Erysipelotrichi. Erysipelotrichales	Control	3.766481
Fusobacteria. Fusobacteriia. Fusobacteriales. Fusobacteriaceae. Fusobacterium	Control	3.705831
Proteobacteria. Gammaproteobacteria. Pseudomonadales	Control	3.685787
Firmicutes. Clostridia. Clostridiales	Control	3.680594
Firmicutes. Clostridia. Clostridiales	Control	3.68059
Firmicutes. Bacilli. Lactobacillales. Lactobacillaceae. Lactobacillus	Control	3.564198

Firmicutes. Bacilli. Lactobacillales. Lactobacillaceae	Control	3.564028
Firmicutes. Clostridia. Clostridiales. Lachnospiraceae	Control	3.533256
Firmicutes. Erysipelotrichi. Erysipelotrichales. Erysipelotrichaceae. Eubacterium	Control	3.449726
Bacteroidetes. Bacteroidia. Bacteroidales. Prevotellaceae. Prevotella	Control	3.410988
Bacteroidetes. Bacteroidia. Bacteroidales. Prevotellaceae	Control	3.410912
Fusobacteria. Fusobacteriia. Fusobacteriales. Fusobacteriaceae	Control	3.395532
Firmicutes. Clostridia. Clostridiales. Lachnospiraceae. Blautia	Control	3.357533
Actinobacteria. Coriobacteriia	Control	3.334641
Firmicutes. Clostridia. Clostridiales. Clostridiaceae. 02d06	Control	3.334051
Actinobacteria. Coriobacteriia. Coriobacteriales	Control	3.332986
Actinobacteria. Coriobacteriia. Coriobacteriales. Coriobacteriaceae	Control	3.331738
Actinobacteria. Coriobacteriia. Coriobacteriales. Coriobacteriaceae. Collinsella	Control	3.30066
Proteobacteria. Gammaproteobacteria. Pasteurellales. Pasteurellaceae.	Control	3.239966
Bacteroidetes. Bacteroidia. Bacteroidales	Control	3.235277
Bacteroidetes. Bacteroidia. Bacteroidales	Control	3.235267
Proteobacteria. Gammaproteobacteria. Pseudomonadales. Moraxellaceae. Enhydrobacter	Control	3.221999
Actinobacteria. Actinobacteria. Actinomycetales. Actinomycetaceae. Actinomyces	Control	3.179384
Actinobacteria. Actinobacteria. Actinomycetales. Actinomycetaceae	Control	3.174711
Firmicutes. Clostridia. Clostridiales. Veillonellaceae	Control	3.161349
Fusobacteria. Fusobacteriia. Fusobacteriales. Fusobacteriaceae. Cetobacterium	Control	3.128093
Firmicutes. Erysipelotrichi. Erysipelotrichales. Erysipelotrichaceae.	Control	3.123658
Firmicutes. Erysipelotrichi. Erysipelotrichales. Erysipelotrichaceae. Catenibacterium	Control	3.107966
Proteobacteria. Gammaproteobacteria. Pseudomonadales. Moraxellaceae. Psychrobacter	Control	3.10006
Firmicutes. Clostridia. Clostridiales. Lachnospiraceae.	Control	3.098178
Proteobacteria. Betaproteobacteria. Burkholderiales. Alcaligenaceae	Control	3.062875
Proteobacteria. Betaproteobacteria. Burkholderiales. Alcaligenaceae. Sutterella	Control	3.061185
Firmicutes. Clostridia. Clostridiales. Ruminococcaceae	Control	3.041962
Firmicutes. Clostridia. Clostridiales. Clostridiaceae.SMB53	Control	3.030355
Firmicutes. Clostridia. Clostridiales. Lachnospiraceae. Dorea	Control	3.010283

Table S4. Relative abundance (relative to staphylococcal sequences) of staphylococcal species.

	Control								Superficial Bacterial Folliculitis												Epidermal Collarette								
	Hgroin	Baxilla	Bgroin	Haxilla	Jaxilla	Jgroin	Raxilla	Rgroin	D4Fol1	D3Fol	D4Fol2	D8Pus	D7Pus1	D1Fol3	D6Fol	D7Pus2	D2Fol	D1Fol1	D1Fol2	D5Fol	D10Col2	D5Col2	D5Col1	D10Col1	D4Col	D9Col1	D6Col	D9Col2	D11Col
S. aureus	0	0	0	0	NA	NA	NA	NA	0	0	0	0	0	0	0	0	0.007	0	0	0	0	0	0	0	0	0	0	0	0
S. capitis	0.002	0	0	0	NA	NA	NA	NA	0	0	0.003	0	0	0	0	0	0	0	0	0	0	0	0	0	0	0.047	0	0	0
S. epidermidis	0	0	0	0.025	NA	NA	NA	NA	0	0	0	0	0	1	0	0	0.117	0	0	0	0	0.064	0	0	0	0	0	0	0
S. pasteuri	0	0	0	0	NA	NA	NA	NA	0	0	0	0	0	0	0	0	0	0	0	0	0	0.467	0	0	0	0	0	0	0
S. pseudintermedius	0.998	1	1	0.975	NA	NA	NA	NA	1	1	0.909	1	1	0	1	1	0.876	0	1	1	1	0.468	1	1	1	0.951	1	1	1
Unclassified	0	0	0	0	NA	NA	NA	NA	0	0	0.089	0	0	0	0	0	0	0	0	0	0	0	0	0	0	0.002	0	0	0
Number of staphylococcal sequences	529	246	81	40	0	0	0	0	9021	4861	2616	6612	891	398	474	448	282	81	23	1	6271	4666	5148	7370	2442	2570	2322	5931	1299

© 2020 by the authors. Submitted for possible open access publication under the terms and conditions of the Creative Commons Attribution (CC BY) license (http://creativecommons.org/licenses/by/4.0/).
